# Racial Categories in Medicine: A Failure of Evidence-Based Practice?

**DOI:** 10.1371/journal.pmed.0040287

**Published:** 2007-09-25

**Authors:** George T. H Ellison, Andrew Smart, Richard Tutton, Simon M Outram, Richard Ashcroft, Paul Martin

## Abstract

Race and ethnicity are imprecise markers of the genotypic and sociocultural determinants of health, argue the authors.

In this issue of *PLoS Medicine,* Lundy Braun and colleagues from the Race, Medicine, and Science Workshop discuss “the trouble with race” [1]. They argue that the heterogeneity of racial and ethnic categories (and a lack of consensus on how these categories should be defined and measured) make them poor markers for underlying differences in the genotypic, cultural, and structural characteristics responsible for inequalities in health (where “structural characteristics” include a diverse range of historical and contemporary socio-political factors responsible for differential access to health resources and differential exposure to health risks). Such racial and ethnic categories, they argue, are also poor markers for the differential efficacy of diagnostic and therapeutic interventions.

However, the authors distinguish between what they feel to be the appropriate *descriptive* use of such categories (to identify differences in health and health care that warrant further investigation and intervention), and their inappropriate *attributive* use (to identify the causal mechanisms involved and to select clinical interventions). To address the latter, they propose substantial improvements in international scientific standards and clinical training. They suggest that these improvements would help to prevent biomedical research and practice from: (1) reifying the discredited notion of races as natural, genetically distinct subspecies; and (2) treating ethnic groups in stereotypical ways as if they were genetically or socio-culturally homogeneous.

These seem entirely sensible recommendations given that the *descriptive* use of racial and ethnic categories to identify differences in health and health care can lead to the inappropriate *attributive* use of disaggregated findings in ways that misidentify the causal mechanisms involved and undermine the impact of training in patient-centred care [2]. However, while it is crucial to address this drift from *description* to *attribution*, our research on the use of racial and ethnic categories in genetics and biomedicine suggests that Braun et al.'s proposals face a number of intractable problems.

**Linked Essay**

This Perspective discusses the following new Essay published in *PLoS Medicine:*

Braun L, Fausto-Sterling A, Fullwiley D, Hammonds EM, Nelson A, et al. (2007) Racial categories in medical practice: How useful are they? PLoS Med 4(9): e271. doi:10.1371/journal.pmed.0040271

In this Essay, the authors address the question of whether it is good medical practice for physicians to “eyeball” a patient's race when assessing their medical status.

## Problems with Definition and Standardisation

In particular, we have found that there is a lack of consensus about what race and ethnicity mean [3] and how these should be operationalised [4]. As a result, researchers and practitioners may conflate the utility of racial and ethnic categories for sampling diverse study populations with their ability to identify and address aetiological variation therein [5]. At the same time, there is widespread concern about the socio-political sensitivity of such categories and a desire to avoid stigmatising populations and groups found to be at greater risk of disease [6]. This concern has led many of the researchers we interviewed to adopt the more socially acceptable term “ethnicity” in preference to “race” [6]. It has also led some researchers to use crude socio-political classifications (such as the categories developed by the United States Office of Management and Budget [OMB] and by the United Kingdom Office for National Statistics [ONS]) not only for sampling diverse study populations and *describing* population differences in health and health care, but also for *attributing* these differences to innate genotypic and/or socio-cultural causes [4].

Thus, even though the researchers themselves often recognise that racial and ethnic categories are imprecise markers of genotypic and socio-cultural determinants of variation in health [5], they have adopted an essentially pragmatic response to the perceived need for standardised, salient, and politically sensitive classifications [4]. Some justify this response on the grounds that however crude racial and ethnic categories might be, they can nonetheless successfully *describe* differences between populations that are biomedically relevant [6]. Others argue that using crude generic socio-political classifications of race and ethnicity (as opposed to more precise bespoke scientific categories) helps facilitate the “portability” and “translation” of findings both within the biomedical research community itself, and between researchers, clinicians, and policy makers [4].

## National Mandates to Describe Race and Ethnicity

The use of crude socio-political categories of race and ethnicity to *describe* variation in health risks and health needs, and to *attribute* these differences to innate genotypic and socio-cultural factors, has a long and discredited history, as Braun and colleagues describe [1]. However, more recently this practice seems to have been resurrected and validated, however unwittingly, by the adoption of such categories to promote equitable participation of “minorities” in research and to ensure that researchers are able to identify and *describe* any differences between racial and ethnic groups that warrant further investigation and intervention [7].

Certainly, some of the US-based researchers we interviewed cited the adoption of the OMB categories by the United States National Institutes of Health (NIH) to justify their use of such categories in their research [6]. In the UK, the Department of Health (DH) has mandated the classification of all National Health Service patients using the ONS categories since 1995 [8]. Although there is currently no requirement for UK-based biomedical scientists to use these categories in their research, we have found that many have adopted the ONS categories in their sampling strategies *and* analytical designs [4]. Indeed, it seems likely that more researchers will follow suit in response to a 2001 directive from the DH which charges researchers to ensure that “the body of research evidence available to policy makers reflects the diversity of the population” [9] and requires research ethics committees to scrutinise the diversity of proposed study populations along these lines [10].

In this way, policies that use crude socio-political categories of race and ethnicity to promote equitable participation in biomedical research, and to provide *descriptive* evidence of variation in health risks and health needs that warrant further investigation and intervention, run the risk of undermining moves to generate the more precise *attributive* evidence required to improve the equitable delivery of health care. This is because, despite growing consensus about the imprecision of racial and ethnic categories as markers for genotypic and socio-cultural determinants of health, the use of these categories to promote equitable participation in biomedical research and to *describe* variation in health risk leads to the use of the same crude categories to (mis)*attribute* causality and thereby (mis)identify health care needs.

## Toward the Use of Context-Specific Attributive Markers

The *attributive* use of crude socio-political categories of race and ethnicity in biomedical research and practice is not, however, an inevitable consequence of policies to promote equitable inclusion in research or to *describe* disparities in health and health care that warrant further investigation and intervention. Researchers are not required to limit the variables they use to explore disparities in health, and clinicians are not obliged to use crude racial and ethnic categories to select the care they provide. But researchers and clinicians do need to be encouraged to use more specific *attributive* markers of genotype, culture, and structural disadvantage wherever appropriate.

For example, in transplantation research, the polymorphic genotypes that determine histocompatibility antigens (HLAs) and influence the success of organ transplants are known to vary in frequency amongst different racial and ethnic groups [11]. These genotypic differences have often been cited to explain racial and ethnic variation in morbidity and mortality amongst patients requiring organ transplantation [12]. Yet these genotypic differences cannot explain differential access to organ transplantation nor the differential efficacy of transplantation practices. Instead, we need to examine the differential impact of cultural and structural factors that influence the availability of suitable organs [11], and of clinical policies that prioritise the transplantation of organs to HLA-matched recipients rather than to recipients at greatest clinical need [13]. Thus, crude socio-political categories of race and ethnicity are only useful as *descriptive* markers of the potential for racial/ethnic discrimination and of related structural factors that influence exposure to health risks, access to health care, and inclusion in health research. And even their apparent utility for this specific purpose is likely to be undermined by the fluid and context-specific nature of racial and ethnic identities, which resist standardisation for use in different scientific, social, and clinical contexts. Indeed, attempts to standardise racial and ethnic categories for use across different national settings have met with little success. Such attempts have concluded that it may only be possible to use context-specific categories to assess discrimination and related structural factors associated with each setting's particular history and contemporary experience of racial and ethnic identities [14].

## A Role for the United States National Institutes of Health and World Health Organization?

So while we would support Braun and colleagues' call for international consensus to improve the use of racial and ethnic categories in biomedical research and practice [1], such categories cannot and should not be standardised for use in all scientific, social, and clinical contexts, even if only for use as *descriptive* variables. Instead, we need to recognise that different racial and ethnic categories are needed to describe inequalities in health and health care in contexts where these have different salience and meaning. At the same time, alternative *attributive* markers of genotypic, cultural, and structural determinants of variation in health and access to health care need to be developed in order to: (1) improve the aetiological precision of biomedical research; and (2) facilitate the translation of research on the causes of variation in health across racial and ethnic groups into appropriate care for individual patients. This more precise approach would help to address a long-standing problem with evidence-based practice, which often struggles to apply the results of epidemiological research on populations to the clinical care of individuals [15].

Certainly, the NIH concedes that “The scientific question being addressed in the study should guide investigators' decisions regarding collection of any additional information on ethnicity and race” [16]. However, our proposal for the use of different racial and ethnic categories as *descriptive* variables in different scientific, clinical, and social contexts, and for more precise genotypic, cultural, and structural variables to *attribute* the causes of racial and ethnic inequalities in health and health care, faces a number of difficult challenges. These include a lack of consensus on whether, and how, race and ethnicity should be operationalised in different scientific, clinical, and social contexts [3,4,14,17], and the need to develop standard instruments for capturing genotypic, cultural, and structural characteristics amenable for use across these contexts. It is also unclear who might be best placed to promote such consensus or develop such instruments. Funding agencies and those generating practice guidelines, such as the NIH and the World Health Organization, have a potential role to play (as Braun and colleagues suggest [1]). But these bodies cannot enforce guidelines for research sponsored by other funders nor guidelines for practice implemented by other health care providers. This would require unprecedented agreement amongst a comprehensive international consortium of funders and providers.

## A Role for Biomedical Journals?

An alternative approach would be to target the gatekeepers of research dissemination, particularly the editors of biomedical journals, who have displayed increasing willingness to improve the use of racial and ethnic categories in published research [18]. Certainly, our research has identified many journals that have generated a range of dedicated guidelines for authors [4,17], even if many biomedical journals simply encourage authors to “justify” the categories they use. These include, for example, the 648 journals signed up to the International Committee of Medical Journal Editors' *Uniform Requirements for Manuscripts Submitted to Biomedical Journals: Writing and Editing for Biomedical Publication,* which recommend that “When authors use variables such as race or ethnicity, they should define how they measured the variables and justify their relevance” [19].

However, we have also found that many editors are unwilling to generate and impose guidelines without the support of the scientific communities they serve [6], and that even when detailed guidelines have been produced these have had little impact on the content of the articles they publish [20]. International consensus amongst biomedical researchers and practitioners is therefore required to support editors in the development and application of guidelines for:

1. improving the use of context-specific racial and ethnic categories as *descriptive* markers of inequalities in health and health care;
2. promoting the use of more precise and portable *attributive* markers of the genotypic, cultural, and structural determinants of racial and ethnic inequalities in health and health care; and thereby
3. generating evidence from population studies of racial and ethnic groups that can be used to improve the care of individual patients from these groups across different social and clinical contexts.

## Abbreviations

DH: United Kingdom Department of Health
HLA: histocompatibility antigen
NIH: United States National Institutes of Health
OMB: United States Office of Management and Budget
ONS: United Kingdom Office for National Statistics
